# A case–control study of risk of leukaemia in relation to mobile phone use

**DOI:** 10.1038/sj.bjc.6605948

**Published:** 2010-10-12

**Authors:** R Cooke, S Laing, A J Swerdlow

**Affiliations:** 1 Section of Epidemiology, Sir Richard Doll Building, Institute of Cancer Research, 15 Cotswold Road, Sutton, Surrey SM2 5NG, UK

**Keywords:** leukaemia, mobile phones, radiofrequency, epidemiology, case–control

## Abstract

**Background:**

Mobile phone use is now ubiquitous, and scientific reviews have recommended research into its relation to leukaemia risk, but no large studies have been conducted.

**Methods:**

In a case–control study in South East England to investigate the relation of acute and non-lymphocytic leukaemia risk to mobile phone use, 806 cases with leukaemia incident 2003–2009 at ages 18–59 years (50% of those identified as eligible) and 585 non-blood relatives as controls (provided by 392 cases) were interviewed about mobile phone use and other potentially aetiological variables.

**Results:**

No association was found between regular mobile phone use and risk of leukaemia (odds ratio (OR)=1.06, 95% confidence interval (CI)=0.76, 1.46). Analyses of risk in relation to years since first use, lifetime years of use, cumulative number of calls and cumulative hours of use produced no significantly raised risks, and there was no evidence of any trends. A non-significantly raised risk was found in people who first used a phone 15 or more years ago (OR=1.87, 95% CI=0.96, 3.63). Separate analyses of analogue and digital phone use and leukaemia subtype produced similar results to those overall.

**Conclusion:**

This study suggests that use of mobile phones does not increase leukaemia risk, although the possibility of an effect after long-term use, while biologically unlikely, remains open.

Use of mobile phones has increased rapidly in recent years, leading to public interest about possible carcinogenic effects. There is no known biological mechanism by which radiofrequency (RF) exposure from mobile phones could cause leukaemia or any other cancer (Scientific Committee on Emerging and Newly Identified Health Risks (SCENIHR), 2009); however, exposure is at least anatomically relevant, as there is considerable bone marrow in the skull and mandible (Ellis, 1961).

Expert groups have recommended that epidemiological studies be conducted on the risks of brain tumours, acoustic neuromas, salivary gland tumours and leukaemia (EC Expert Group, 1996; Independent Expert Group on Mobile Phones (IEGMP), 2000). The first three of these have been addressed by several studies, but have not provided persuasive evidence of an association (International Commission on Non-Ionizing Radiation Protection (ICNIRP), 2009; The INTERPHONE Study Group, 2010). Leukaemia is of particular interest because it has a shorter induction period than solid cancers after other exposures – risk increases 1–2 years after exposure to ionising radiation or alkylating chemotherapy and reaches a peak at 5–9 years, whereas solid tumour risk begins to increase 5–10 years after exposure and remains raised for at least 25 years (van Leeuwen and Travis, 2005); an earlier effect of mobile phone use on leukaemia risk than for solid tumours is, therefore, plausible.

There have been few studies of mobile phone use and leukaemia (Dreyer *et al* 1999; Schüz *et al*, 2006; Kaufman *et al*, 2009), while studies on leukaemia risk after RF exposure from other sources have given inconsistent results (Ahlbom *et al*, 2004; Scientific Committee on Emerging and Newly Identified Health Risks (SCENIHR), 2009). We, therefore, conducted a case–control study with over 800 cases in South East England to investigate leukaemia risk in relation to mobile phone use.

## Materials and methods

A case–control study was conducted in South East England (London and the surrounding counties). Cases were defined as people diagnosed with leukaemia (excluding chronic lymphocytic leukaemia, as past evidence suggests that this has a different aetiology from other subtypes (Boice, 2006)) at ages 18–59 years, who were resident in the study region at diagnosis, and diagnosed within a specified time period – namely 2003–2007 in most of the region and 2003–2009 in two areas.

Cases were mainly ascertained via healthcare staff in haematology and oncology units. Contact was made approximately weekly so that potential cases could be identified rapidly, minimising the number unable to participate because of illness or death. Listings of leukaemia diagnoses were also obtained from the Thames Cancer Registry at least every 6 months, and to ensure completeness, extra cases identified from these listings were included as potential cases, once the relevant hospital contact had confirmed their eligibility. Potential cases who were hospital in-patients were invited to participate in the study in person by a research nurse, after clinical staff had confirmed that it was appropriate to approach them. Potential cases who were at home were invited to participate by letter, and later contacted by telephone by a research nurse if they did not reply. The information provided to potential participants presented the study as an investigation of the relation of features of modern lifestyle to the causation of leukaemia; it did not specify mobile phone use as the main exposure of interest, mentioning this only as one of the several subjects that would be covered.

Controls were non-blood relatives of the cases (or equivalent for non-married couples), who had never been diagnosed with leukaemia, did not live with the case and fitted the same age and residence criteria as the cases. This control source was used because it has become increasingly difficult to obtain high response rates for population-based controls (Morton *et al*, 2006). It was hoped that people with a relative with leukaemia would be more motivated to participate in the study, and that this would result in higher response rates. Cases were asked to provide details of all non-blood relatives, and those who met the inclusion criteria and who the case was willing for us to approach were invited to participate by letter, which was followed up by phone by a research nurse, if they did not reply.

No information on mobile phone use of non-participants was collected from healthcare staff or relatives, as ethics permission was not obtained for this.

Participants were interviewed face-to-face by a research nurse in hospital, at home or at another convenient place and signed informed consent was obtained. Medical research ethics approval was granted by the South East Multi-centre Research Ethics Committee. Participants were asked about demographic factors and exposure to a range of known and potential risk factors for leukaemia, including RF exposure, with a particular focus on mobile phone use. The phone section of the questionnaire was based on that used in the Interphone study (Cardis *et al*, 2007), with some modifications. Subjects were asked whether they had ever used a mobile phone regularly, which was defined as at least 6 months making or receiving at least one call a week on average. They were asked which makes and models they had used and to specify, for each phone, the year that they started and stopped using the phone, their average number of calls per day or per week, the average length of the calls, the phone network used and the proportion of use that was hands free. If participants were unable to provide an average call frequency or duration, they were asked to give a range. Data on use of cordless phones were not collected, as the power output from these is much lower than that from mobile phones – around 10% of the typical output from a digital mobile phone and 1% of that from an analogue phone (Advisory Group on Non-Ionising Radiation (AGNIR), 2003; Hardell *et al*, 2006b).

### Statistical analysis

Analyses were conducted for leukaemia overall, and separately for acute myeloid leukaemia (AML), acute lymphoblastic leukaemia (ALL) and chronic myeloid leukaemia (CML). All controls were used in all analyses. Exposures were evaluated up to the diagnosis date for cases, and an equivalent date before the interview for controls, referred to as the ‘reference date’, so that phone use in controls was not overestimated because of rapidly increasing use in the general population during the study period and controls being interviewed later than cases, and so that similarly distant time periods were recalled by cases and controls. As controls were not individually matched to cases, the reference dates for controls were constructed by stratifying cases by half-year interview period and whole-year interview lag time, and randomly allocating controls in each half-year interview period to lag time strata in the same proportions as the cases, so that the distributions of cases and controls over the lag time strata were similar. The mean lag time for cases in each stratum was subtracted from the interview date for each control in the same stratum to create their reference date.

The phone use histories provided by participants were used to calculate number of years since first use of a mobile phone, total lifetime years of use and total cumulative number of calls and hours of use. Adjusted versions of these variables were calculated taking into account the extent of hands-free use, by multiplying the average call length for each phone by the proportion of time that the phone was not used hands free. Participants’ cumulative number of calls or hours of use were categorised as unknown if the extent of usage of at least one phone was unknown. As this meant that participants who had used more phones might be more likely to be in the unknown category, however, we also re-analysed the data after imputing missing phone usage values by using data from the previous or next mobile phone the participant used, where this information was available. Where a participant gave a range for their average call frequency or duration, the mean of the upper and lower bounds was used in calculations. These analyses were repeated, however, using a number of different methods of imputing the value from a range, to assess whether our choice of method had influenced the results. In these alternative methods, the value was imputed as the lower bound, the upper bound and weighted means giving more weight to the lower, or upper, bound. Analyses were also repeated classifying the value as unknown where a range was given.

Risks of leukaemia in relation to aspects of phone use were estimated by calculating odds ratios (OR) and 95% confidence intervals (CI), using unconditional logistic regression adjusted for 5-year age group at reference date, sex, socio-economic status (classified using the residentially based ACORN score (CACI, 2009)), area of residence (London or surrounding area), ethnicity (white, non-white or mixed or unknown), smoking status at reference date (current smoker, ex-smoker, never smoker or unknown) and interview lag time and half-year period stratum. Analyses were also repeated using conditional logistic regression, with strata of age, sex and area of residence and adjusted for socio-economic status, ethnicity, smoking status and interview lag time and half-year period stratum. Trend tests were conducted for ordinal variables, using a likelihood-ratio test comparing a model excluding the variable of interest with a model containing the variable, as a categorical variable with each category's value being the mean of the values in that category.

The above analyses were repeated for analogue and digital phone use separately and by sex. We also re-analysed the data excluding participants who had been exposed to leukemogenic chemotherapy, radiotherapy or chloramphenicol, or who had a cytogenetic abnormality predisposing to leukaemia, adjusting for reported use of other communication devices and occupational exposure to radar, telecommunication antennae and masts, and excluding participants who reported implausibly high levels of phone use. Analyses were conducted using the statistical package Stata (Stata Corporation, 2007).

## Results

We identified 1660 potentially eligible leukaemia cases, of whom 380 had died; 52 were too ill or otherwise medically unsuitable to be interviewed; for 54 cases, we could not gain consultant permission; for 33 cases, ascertained via the cancer registry, the hospital concerned had no record of the patient attending for leukaemia; 47 cases were not approached for other reasons; 176 cases replied to the invitation letter to say that they were not willing to participate; 112 cases never replied and could not be contacted by phone and 806 cases were interviewed. The cases who took part comprised 50% of the eligible leukaemia cases identified (excluding the 33 patients who were not recorded as leukaemia patients at the relevant hospitals), and 82% of those who were sent an invitation to take part and are known to have received it. Participation rates were similar for males (49%) and females (50%); diminished with lower socio-economic status (62% in the highest group, 34% in the lowest) and diminished with calendar year (54% in 2003, 44% in 2008/2009). The interviewed cases comprised 449 with AML, 125 with ALL, 154 with CML, 55 with hairy cell leukaemia, 7 with acute monocytic leukaemia and 16 other types or of mixed phenotype (Table 1).

Of the interviewed cases, 57% provided details of eligible relatives, 28% had no suitable relatives and 15% were not willing to provide details of relatives. In total, 781 potential controls were identified and invited to take part, of whom 589 (75%) were interviewed (4 were aged over 59 years at reference date, and were, therefore, excluded from analysis). This comprised 75% of all those who sent an invitation to take part, and 86% of those who were known to have received the invitation letter. The participation rate was slightly higher in females (77%) than males (73%), diminished with decreasing socio-economic status (80% in the highest group, 71% in the lowest), and decreased with calendar year (81% in 2003, 66% in 2008/2009) and younger age (>90% at ages >50 years, 60% at ages <25 years). The interviewed controls were provided by 392 different cases, and comprised 203 sisters-in-law (or equivalent for non-married couples), 175 brothers-in-law (or equivalent), 50 sons-in-law (or equivalent), 43 daughters-in-law (or equivalent) and 118 other non-blood relatives.

Regular phone users had a similar risk of leukaemia to never regular users (OR=1.06, 95% CI=0.76, 1.46; Table 2). There was no significant trend of risk in relation to years since first use or total years of use, although the risk was non-significantly raised in the longest categories (OR=1.87, 95% CI=0.96, 3.63 and OR=1.63, 95% CI=0.81, 3.28, respectively).

Risk was not related to cumulative number of calls or hours of use. The results were similar when analyses were repeated using different methods of imputing average frequency or length of calls from a range (not in table). Repetition of the above analyses separately by digital or analogue phone use (Table 3) and by sex (not in table) gave similar results.

In analyses by cytological subtype (Table 4), there was no significant variation in results by type, and no significant trend in risk with years since first use, years of use or cumulative hours of use. The only significant result was a reduced risk of ALL 10–14 years after first use (OR=0.37, 95% CI=0.14, 0.94). There was a borderline significantly raised risk of AML ⩾15 years after first use (OR=2.08, 95% CI=0.98, 4.39) and of ALL after 10–14 years of use (OR=0.39, 95% CI=0.15, 1.00). The results were unchanged when the 36 cases and 9 controls who had been exposed to leukemogenic chemotherapy, radiotherapy or chloramphenicol or who had a cytogenetic abnormality predisposing to leukaemia were removed. Adjusting for the extent of hands-free use, or for other RF exposure through use of other communication devices or occupational exposure, did not materially alter the results, and similar results were obtained using conditional logistic regression (not in table). In analyses based on means where ranges were given, 26 (3.2%) cases and 17 (2.8%) controls were allocated values of average daily use of a particular phone of over 6 h by the calculations, and 83 (10.3%) cases and 53 (8.8%) controls were allocated values of over 2 h. Re-analysing the data after excluding participants with a calculated usage of over 2 h per day, as well as those who reported first using a mobile phone before 1985, when the first mobile phone networks became available in Britain (4 cases, 2 controls), produced similar results to those in Table 2 (not in table). Repeating the analysis restricted to the highest socio-economic status category, in which the response rate was highest, produced no significant results, and no evidence of any raised risks (not in table).

## Discussion

There are currently >4.6 billion mobile phone subscriptions in use worldwide (International Telecommunication Union (ITU), 2010), and thus any potential health effects could be of great public health importance. Although no biological mechanism has been identified, there is considerable public and scientific interest in the possibility that RF exposure from phone use might affect cancer risk (Scientific Committee on Emerging and Newly Identified Health Risks (SCENIHR), 2009). Leukaemia risk is of interest because of the proximity of phone use to the skull and mandible, which contain 13% of the body's active bone marrow (Ellis, 1961). We found no association between regular phone use and developing leukaemia, and no significant results relating to time since first use, total years of use or cumulative number of calls or hours of use, although there was a non-significantly 1.9-fold raised risk in people who had first used a mobile phone 15 or more years before the reference date.

The results from the few previous studies are largely consistent with ours. Two cohort studies based on subscription data, and hence without direct information on who used the phone, one in Denmark with 351 leukaemia cases (Schüz *et al*, 2006) and one in the United States with 15 deaths from leukaemia (Dreyer *et al*, 1999), found no raised risk in phone subscribers. A case–control study of 180 cases in Thailand (Kaufman *et al*, 2009), among participants with a low prevalence of phone use, found a slight non-significantly raised risk in people who had ever used a mobile phone. There are no previous data on risk after 15 years of use, but the Danish cohort, in which the longest subscription category was 10 or more years, found no substantially raised risk and no evidence of a trend. It seems unlikely that the raised risk in our study represents a true effect, as leukaemia is associated with particularly short induction periods for known carcinogens, for example ionising radiation and alkylating chemotherapy (van Leeuwen and Travis, 2005) – whereas there was no evidence of a raised risk at shorter times since first use in our data or the Danish cohort.

For both cumulative number of calls and hours of use, the highest risk was found in the unknown category. As people became more likely to be in this category the more phones they had used, this could suggest that more cases than controls had used a large number of phones. However, a re-analysis of these variables imputing missing phone usage information, where possible, produced similar results, suggesting that this is not the reason, and that cases simply found it harder than controls to recall details of their phone use. As participants often gave a range when reporting their average frequency and length of calls, and there is no obviously correct method here, we repeated the analysis several times using different methods. We also repeated analyses excluding participants who reported implausible phone usage patterns or dates. The results from these analyses were similar to those in Table 2.

The three main leukaemia subtypes share some risk factors, for example ionising radiation (Preston *et al*, 1994), but other exposures have different effects on different subtypes, for example smoking (Doll, 1996). We, therefore, analysed the effect of phone use on each subtype separately. The only previous such analysis was based on <20 ever users per subtype and found no significant results (Kaufman *et al*, 2009). Our analyses produced similar results to the combined analyses; the only significant result was a reduced risk of ALL in the 10–14 years since first use category, with a near-significant reduced risk in the corresponding category of lifetime years of use; however, these were based on small numbers, and there was no evidence of a trend in either variable. We also found a near-significant raised risk of AML in the highest category of years since first use, but no trend and as discussed above, AML tends to have a short latency period following other risk factors, so this is unlikely to represent a true effect. As we conducted a large number of comparisons in this analysis, one might expect some significantly reduced or raised risks to occur by chance alone.

If phone use had a leukemogenic effect, one might expect this to be greatest in relation to use of analogue phones, as these have a higher average power output than digital phones (Ahlbom *et al*, 2004). However, we saw neither raised risk in regular users of either type, nor any association between risk and years since first use, total years of use or total hours of use of either type.

As the analyses in this study are based on self-reported data, and participants’ first mobile phone use was often many years before their interview (15 years or more for 81 participants), it seems likely that inaccuracies in recall will have led to a substantial amount of misclassification. Investigations into the validity of phone use data within the Interphone study (Cardis *et al*, 2007), for which phone use histories were collected in a similar way to ours, found substantial random error and some systematic error in reported mobile phone use, with participants tending to underestimate number of calls and overestimate length of calls (Vrijheid *et al*, 2006). If there were non-differential misclassification, this would be expected to bias results towards the null. A possible increase in overestimation of phone use in more distant time periods by cases was also found in Interphone (Vrijheid *et al*, 2009a), which could potentially explain the slightly raised risk we found in the highest category of cumulative hours of phone use (Table 2).

Population-based control participation rates have been declining in recent years (Morton *et al*, 2006). We, therefore, used a different control source in this study, non-blood relatives of cases, and achieved a good participation rate (75%). A disadvantage of this control source is potential overmatching, but this would give a risk of loss of statistical efficiency rather than bias (Rothman and Greenland, 1998). On the other hand, the risk of selection bias was reduced by the high participation rate. This may be why we did not find, as in many population-based case–control studies of brain tumour risk in phone users, an apparently reduced risk in users (Schoemaker *et al*, 2005; Lönn *et al*, 2006; Lahkola *et al*, 2007; The INTERPHONE Study Group, 2010), which has been ascribed to potential controls being less likely to participate if they were not regular users (Lahkola *et al*, 2005; Vrijheid *et al*, 2009b). A second reason why our recruitment may not have been biased in this respect is that our recruitment literature described a study of leukaemia and modern life, not specifically of phone use.

Our participation rate among cases identified as eligible was 50%. The main reason for non-participation was not unwillingness to take part, but rather that cases were too ill, or died soon after diagnosis. We made efforts to reduce non-response by using a rapid case ascertainment system, and making follow-up telephone calls to potential participants who did not reply to the invitation letter, which resulted in a participation rate of 82% among those known to have received the invitation to take part, but given the rapid clinical deterioration in many patients, some non-participation was inevitable. If any leukaemias induced by phone use were particularly aggressive, then non-participation because of illness or death could in principle result in any true leukaemogenic effect being underestimated. A number of patients (all 36 CML patients at one hospital) also had to be excluded because they were under the care of a clinician who refused access to her patients; however, this seems unlikely to have caused bias. To investigate the effect of the participation rate, the analysis was repeated, restricted to the highest socio-economic category, in which the response rate was highest, but no evidence of a raised risk was found.

Previous epidemiological studies of the carcinogenicity of mobile phone use have mainly focused on brain tumours, but only those from one group have suggested raised risk in users (Hardell *et al*, 2006a); literature overall suggests no causal association (International Commission on Non-Ionizing Radiation Protection (ICNIRP), 2009). Furthermore, biological studies have found no persuasive evidence of any mechanism by which RF could cause cancers (Scientific Committee on Emerging and Newly Identified Health Risks (SCENIHR), 2009).

In conclusion, our results do not suggest that use of mobile phones increases risk of leukaemia overall, or of any subtype. However, the possibility of an effect after long-term use, although biologically unlikely, is left open, and future results from existing cohorts (and from case–control studies, if any are conducted) would be of interest.

## Figures and Tables

**Table 1:** Leukaemia cases and controls: descriptive characteristics

	**Cases**		**Controls**	
**Characteristic**	**No.**	**%**	**No.**	**%**
*Sex*				
Male	454	56.3	259	44.3
Female	352	43.7	326	55.7
				
*Age at diagnosis (years)* a				
<25	65	8.1	29	5.0
25–34	118	14.6	153	26.2
35–44	218	27.0	164	28.0
45–54	251	31.1	174	29.7
55–59	154	19.1	65	11.1
				
*Socio-economic status (ACORN score)* b				
1–12	198	24.6	136	23.3
13–23	211	26.2	111	19.0
III	209	25.9	183	31.3
IV	96	11.9	85	14.5
V	92	11.4	69	11.8
				
*Place of residence*				
London	445	55.2	283	48.4
Outside London	361	44.8	302	51.6
				
*Ethnicity*				
White	698	86.6	544	93.0
Non-white	100	12.4	32	5.5
Mixed and unknown	8	1.0	9	1.5
				
*Diagnosis*				
AML	449	55.7		
ALL	125	15.5		
CML	154	19.1		
HCL	55	6.8		
Other and unspecified	23	2.9		
Total	806	100.0	585	100.0

Abbreviations: AML=acute myeloid leukaemia; ALL=acute lymphoblastic leukaemia; CML=chronic myeloid leukaemia; HCL=hairy cell leukaemia.

a For controls, age at reference date – see Materials and methods.

b One missing value for socio-economic status, controls.

**Table 2:** Risks of leukaemia in relation to use of mobile phones

	**Cases no.**	**Controls no.**	**Odds ratio** a	**95% CI**
*Ever use* b				
Never or non-regular use	132	99	1.00	
Regular use	674	486	1.06	0.76, 1.46
				
*Years since first use*				
Never or non-regular use	132	99	1.00	
0.5–4^c^	195	146	0.98	0.68, 1.42
5–9	307	229	1.07	0.75, 1.53
10–14	111	89	0.98	0.63, 1.51
⩾15	50	16	1.87	0.96, 3.63
Unknown	11	6	1.08	0.35, 3.39
*P-*trend			0.27	
				
*Lifetime years of use*				
Never or non-regular use	132	99	1.00	
0.5–4^c^	201	153	0.97	0.67, 1.39
5–9	309	228	1.10	0.77, 1.58
10–14	110	84	1.04	0.67, 1.61
⩾15	42	15	1.63	0.81, 3.28
Unknown	12	6	1.17	0.38, 3.61
*P-*trend			0.30	
				
*Cumulative no. of calls*				
Never or non-regular use	132	99	1.00	
<Median (<5350)	292	228	1.01	0.71, 1.42
Median-third quartile (5350–16062)	166	115	1.13	0.76, 1.69
>Third quartile (>16062)	160	113	1.03	0.68, 1.56
Unknown	56	30	1.37	0.78, 2.43
*P-*trend			0.99	
				
*Cumulative hours of use*				
Never or non-regular use	132	99	1.00	
<Median (<284)	283	226	0.97	0.69, 1.36
Median-third quartile (284–1156)	160	115	1.14	0.76, 1.71
>Third quartile (>1156)	176	114	1.19	0.79, 1.80
Unknown	55	31	1.30	0.74, 2.29
*P-*trend			0.31	

Abbreviation: CI=confidence interval.

a Adjusted for age, sex, socio-economic status, area of residence, ethnicity, smoking status and interview lag time and period.

b At least one call a week on average for at least 6 months.

c Lower limit 0.5 years due to definition of regular use.

**Table 3:** Risks of leukaemia in relation to various characteristics of analogue or digital phone use

	**Type of phone**							
	**Analogue**				**Digital**			
**Factor**	**Cases no.**	**Controls no.**	**OR**	**95% CI**	**Cases no.**	**Controls no.**	**OR**	**95% CI**
*Ever use*								
Never or non-regular phone use	132	99	1.00		132	99	1.00	
Regular use of specified type of phone	129	100	1.18	0.75, 1.86	644	476	1.03	0.75, 1.43
Unknown	111	57	1.40	0.85, 2.30	30	10	2.25	0.99, 5.01
								
*Years since first use*								
Never or non-regular phone use	132	99	1.00		132	99	1.00	
0.5–4	1	0			214	157	1.03	0.71, 1.48
5–9	47	51	0.85	0.48, 1.49	285	221	1.02	0.71, 1.47
⩾10	80	47	1.54	0.91, 2.62	46	40	0.78	0.44, 1.38
Unknown	112	59	1.40	0.85, 2.30	129	68	1.35	0.87, 2.08
*P-*trend			0.08				0.65	
								
*Lifetime years of use*								
Never or non-regular phone use	132	99	1.00		132	99	1.00	
0.5–4	72	63	1.08	0.64, 1.80	216	162	1.00	0.70, 1.44
5–9	33	23	1.19	0.60, 2.35	285	214	1.06	0.74, 1.53
⩾10	3	1	5.30	0.30, 94.18	39	38	0.67	0.37, 1.23
Unknown	132	70	1.40	0.88, 2.24	134	72	1.33	0.87, 2.05
*P-*trend			0.25				0.60	
								
*Cumulative hours of use* a								
Never or non-regular phone use	132	99	1.00		132	99	1.00	
<Median	43	42	0.94	0.53, 1.68	260	207	0.98	0.69, 1.38
Median-third quartile	31	24	1.16	0.59, 2.28	133	102	1.05	0.69, 1.59
>Third quartile	32	21	1.42	0.69, 2.93	132	96	1.05	0.68, 1.62
Unknown	134	70	1.45	0.91, 2.32	149	81	1.33	0.87, 2.02
*P-*trend			0.14				0.63	

Abbreviations: OR=odds ratio; CI=confidence interval.

a Analogue: median=129, third quartile=667; digital: median=251, third quartile=1079.

**Table 4:** Risks of acute myeloid leukaemia, acute lymphoblastic leukaemia and chronic myeloid leukaemia in relation to mobile phone use

	**Leukaemia subtype** a								
	**AML**			**ALL**			**CML**		
**Factor**	**Cases no.**	**OR**	**95% CI**	**Cases no.**	**OR**	**95% CI**	**Cases no.**	**OR**	**95% CI**
*Ever use*									
Never or non-regular use	73	1.00		18	1.00		25	1.00	
Regular use	376	1.07	0.73, 1.57	107	0.59	0.31, 1.13	129	1.48	0.85, 2.59
									
*Years since first use*									
Never or non-regular use	73	1.00		18	1.00		25	1.00	
0.5–4	109	1.02	0.66, 1.58	29	0.51	0.24, 1.11	40	1.36	0.72, 2.57
5–9	171	1.08	0.71, 1.65	56	0.64	0.32, 1.30	56	1.56	0.84, 2.90
10–14	58	0.91	0.54, 1.54	12	0.37	0.14, 0.94	28	1.99	0.94, 4.22
⩾15	31	2.08	0.98, 4.39	8	1.41	0.45, 4.37	4	1.25	0.34, 4.65
Unknown	7	0.92	0.26, 3.20	2	0.90	0.12, 6.87	1	0.49	0.02, 9.82
*P-*trend		0.32			0.46			0.34	
									
*Lifetime years of use*									
Never or non-regular use	73	1.00		18	1.00		25	1.00	
0.5–4	113	1.00	0.65, 1.54	30	0.51	0.23, 1.09	40	1.29	0.68, 2.43
5–9	172	1.11	0.73, 1.70	56	0.66	0.33, 1.34	58	1.66	0.89, 3.09
10–14	57	0.98	0.58, 1.65	12	0.39	0.15, 1.00	27	1.97	0.93, 4.21
⩾15	26	1.76	0.81, 3.81	7	1.17	0.36, 3.78	3	1.06	0.25, 4.52
Unknown	8	1.05	0.31, 3.53	2	0.88	0.11, 6.76	1	0.49	0.02, 9.93
*P-*trend		0.39			0.56			0.30	
									
*Cumulative hours of use*									
Never or non-regular use	73	1.00		18	1.00		25	1.00	
<Median	152	0.96	0.64, 1.44	42	0.61	0.30, 1.22	62	1.48	0.82, 2.67
Median-third quartile	92	1.26	0.78, 2.04	25	0.52	0.23, 1.15	32	1.70	0.85, 3.40
>Third quartile	99	1.23	0.76, 2.01	31	0.59	0.27, 1.31	28	1.51	0.72, 3.14
Unknown	33	1.22	0.64, 2.34	9	0.73	0.25, 2.15	7	0.87	0.28, 2.75
*P-*trend		0.48			0.92			0.90	

Abbreviations: AML=acute myeloid leukaemia; ALL=acute lymphoblastic leukaemia; CML=chronic myeloid leukaemia; OR=odds ratio; CI=confidence interval.

a Control numbers as shown in Table 2.
